# Knowledge, Awareness and Practice of Health care Professionals amid SARS-CoV-2, Corona Virus Disease Outbreak

**DOI:** 10.12669/pjms.36.COVID19-S4.2704

**Published:** 2020-05

**Authors:** Naseer Ahmed, Maria Shakoor, Fahim Vohra, Tariq Abduljabbar, Quratulain Mariam, Mariam Abdul Rehman

**Affiliations:** 1 Naseer Ahmed Associate Professor, Department of Prosthodontics, Altamash Institute of Dental Medicine, Karachi 75500, Pakistan; 2 Maria Shakoor Assistant Professor, Department of Prosthodontics, Altamash Institute of Dental Medicine, Karachi 75500, Pakistan; 3 Fahim Vohra Professor, Department of Prosthetic Dental Science, College of Dentistry, King Saud University, Riyadh 11545, Saudi Arabia; 4 Tariq Abduljabbar Professor, Department of Prosthetic Dental Science, College of Dentistry, King Saud University, Riyadh 11545, Saudi Arabia; 5 Quratulain Mariam Lecturer, Department of Prosthodontics, Altamash Institute of Dental Medicine, Karachi 75500, Pakistan; 6 Mariam Abdul Rehman Lecturer, Department of Prosthodontics, Altamash Institute of Dental Medicine, Karachi 75500, Pakistan

**Keywords:** COVID-19, Health care professionals, Knowledge, Awareness, Practice, SARS-COV 2

## Abstract

**Objective:**

To assess the knowledge, awareness and practice level of health care workers towards Corona Virus disease - 2019 (COVID-19).

**Methods:**

A cross sectional study was conducted by administering a well-structured questionnaire comprising of three sections including knowledge, attitude and practice amongst health care professionals in various hospitals and clinics, over a duration of two months ‘Feb-March’ 2020. The data from 810 participants were collected manually as well as through online survey registered on www.surveys.google.com, using a validated questionnaire. The questionnaire comprised of three sections assessing knowledge, awareness and practice of participants. The descriptive analysis was carried out for demographics and dependent variables with statistical program for social sciences. Spearman test was used to detect any relationship between the health care professional response with respect to their gender and level of education. A p value of < 0.05 was considered statistically significant.

**Results:**

More than half (57.2%) of the health care professionals were working in a hospital setting. Fifty two percent of health care professionals had awareness and 72% were practicing adequate measures to combat COVID-19. The majority (81.9%) believed that the sign and symptoms are similar to a common flu and the main strata of population that could be affected by COVID-19 are elderly (79%). Seventy three percent of participants did not attend any lecture, workshop or seminar on COVID-19 for awareness purpose. Sixty seven percent of health care professionals were practicing universal precaution for infection control and 57.4% were using sodium hypochlorite as a surface disinfectant in dental surgeries. There was no significant relationship (p > 0.05) between the health care professionals’ responses with gender and their education level.

**Conclusion:**

The study suggests that the vast majority of the health care professionals have adequate knowledge and awareness related to COVID-19. However some aspects of practice of health care professionals were found to be deficient including, following CDC guidelines during patient care, acquiring verified knowledge related to COVID-19, disinfection protocol and the use of N-95 mask. Mandatory Continued professional development programs including lectures and workshops on COVID-19 for all health care professionals are the need of the hour, to manage the pandemic and limiting the morbidity and mortality related to it.

## INTRODUCTION

CoronaVirus disease 2019 (COVID-19) is a highly contagious viral pandemic affecting more than one million people in more than 200 countries around the globe. It was declared as a public health emergency of international concern (PHEIC) by WHO in Jan’2020. It is caused by a new strain of novel coronavirus (SARS-COV-2) and was first reported in December 2019 in the Chinese province of Wuhan.^1^ The main route of human-to-human transmission is through respiratory droplets (coughing and sneezing). It is also transmitted from contact (shaking hands) with an infected person or a contaminated surface and transferring it to the mouth, nose or eyes.^2^ COVID-19 has an extended incubation period of 2-14 days^3^, with variable severity from asymptomatic to life threatening symptoms among different individuals.^4^ Infected individuals can be asymptomatic, therefore not just making the diagnosis difficult but preventing the transmission of the disease an arduous challenge.^5^-^7^

COVID-19 is reported to manifest clinically with one or more mild symptoms of fever, cough, headache, body aches, dyspnea and fatigue, with symptomatic recovery within a few weeks.^1^ While severely affected individuals show manifestation of progressive respiratory distress syndrome due to the lung substance damage and edematous changes caused by the virus in leading to shock and death in some cases.^8^ The mortality rate range is 0.8 to 4.3% based on the early data from different regions.^9^ A majority of middle to elderly patients with comorbidities including, tumor, cirrhosis, hypertension, coronary heart disease and diabetes are reported dead as a result of COVID-19 infections.^10^ However the data available regarding risk factors of COVID-19 is still preliminary.

To date, no specific antiviral treatment has been confirmed to be effective and no vaccines has been developed for its prevention.^10^ The disease is managed with appropriate symptomatic treatment (antipyretics, analgesics) and supportive care along with training for prevention and control with isolation, and disinfection. The best prevention is to avoid being exposed to the virus 11. Infection preventive and control (IPC) measures that may reduce the risk of exposure include use of face masks; covering coughs and sneezes with a flexed elbow; regular hand washing with soap or disinfection with hand sanitizer containing at least 70% alcohol; maintaining a distance of 1.5 to 2 meters from humans; and refraining from touching eyes, nose, and mouth with hands.^11^

Health care professionals (HCP) including nurses, doctors, intensivist, paramedics, dentist and other hospital staff are playing a critical role throughout the world in combating, preventing and managing patients affected by COVID-19.^11^ However multiple reports of infection and fatalities of HCPs have surfaced in the last few weeks, which are of grave concern.^12^ Awareness, knowledge and practice attitudes among HCPs are critical in the management of viral diseases^13^ and their role in this pandemic is no different. Due to the reported infection rates, it was hypothesized that the knowledge and practice of HCPs related to COVID-19 will be deficient. To prevent COVID-19 infection and disease among HCPs the primary steps should involve establishing an estimate of their knowledge, awareness and practice towards the COVID-19 infections, in addition to an awareness and education program with updated evidence. Therefore, the present study aimed to evaluate the knowledge, awareness and practice towards COVID-19 infection and disease among health care professionals (doctors, dentist, nurses, assistants, technicians and paramedics).

## METHOD

The ethics and review committee of AIDM (EC/02/2020/01) reviewed and approved the project. This cross-sectional study was carried out on Health Care Professionals (HCP) working at different hospitals and clinics, including both the public and private sector. A total of 1000 HCPs including doctors from the medical and dental fields alongside nurses, medical and dental assistants, paramedics and laboratory technicians were approached in the study from February 2020 to March 2020. A consent statement for voluntary participations was included in the questionnaire for all subjects to understand prior to their agreement. Data was collected using a well-structured questionnaire that comprised of 31 pre-defined responses including the demographic, knowledge (K), awareness (A) and practice (P) assessment sections. A group of medical and dental academic staff members not involved in this research, evaluated the content and validity of the questionnaire employed to evaluate the KAP levels of HCPs. The consistency of response evaluated by Cronbach’s alpha was 0.71.

The first section of the questionnaire comprised of six closed ended questions about the demographic details along with education levels, designation, type of workplace and working sector of participants. Section two, had a total of eleven questions focused on knowledge level of health care professional. These included knowledge regarding the awareness, etiology, mode of transmission, incubation period, sign and symptoms, duration of presence outside the human body, age group of individuals, mortality rate, ways of prevention and treatment options currently available for COVID-19. The third section of questionnaire had fourteen questions with regards to attitude and practice of HCP’s which comprised of questions related to contagious nature, prevention of spread, animal to human transmission, reinfection rate in humans, availability of vaccine, community threats, practice of CDC guidelines and patient care. The third section also included encountering of patient, lectures and workshops for knowledge of COVID-19, washing of hands before and after patient treatment, following universal precautions for infection control and use of surface disinfectant to contain and eliminate virus clusters of COVID-19.

The questionnaire was distributed in two phases; a manual phase in which 300 printed questionnaires were hand distributed and responses were recorded by non-probability convenience sampling method. In order to gain an understanding of how much COVID-19 was understood amongst health care professionals at the time of global pandemic. The survey questionnaire was administered to the HCPs serving at various medical and dental centers. In the online phase, 700 questionnaires were uploaded and disseminated through Google Surveys by forwarding web page links through emails. The questionnaire link was also distributed through social media outlets including, WhatsApp, Facebook, and Twitter. No obligation was placed, and all participants were kept anonymous. Online response record, online survey link was available for the complete study duration.

The data collected was analyzed through Statistical Package for Social Sciences (SPSS-Version 25). Descriptive statistics and spearman correlation were performed, considering p < 0.05 as statistically significant.

## RESULTS

A total of 810 HCPs at a response rate of 81% completed the questionnaire. One hundred and ninety eight hand distributed and 612 questionnaires sent through digital portals were completed by participants. The descriptive data of the participants is presented in Table-I. Out of the total 810 participants, 388 were males and 422 females with a common age range of 21.2 to 30.8 years (69%). Among the included participants, 400 were dentist, 215 were medical doctors and 195 hospital auxiliary staff i.e. technicians, hygienist, nurses, and assistants. By designation, 431 were general medical or dental professionals and 226 were specialist’s and consultants. 545 HCPs belonged to private and 265 worked at government, primary care or specialist centers and hospitals. Majority of participants, 464 were working in hospital while 346 were in clinics. The present study reported no significant relationship between the dental care professionals’ responses with gender and their education level (p<0.05).

**Table-I:** Descriptive statistics (n=810).

		Number and Percentage
Age	22-25 years	300 (37)
	26-30 years	259 (32)
	31-35 years	19 (2.4)
	36-40 years	13 (1.7)
	41-45 years	101 (12.4)
	Above 45 years	118(14.5)
Gender	Male	388 (47.9)
	Female	422 (52.1)
Education	BDS	400 (49.3)
	MBBS	215 (26.5)
	Others	195 (24)
Qualification	Graduate	431 (53.2)
	Postgraduate	226 (27.9)
	Others	153 (18.8)
Workplace	Hospital	464 (57.2)
	Clinic	346 (42.7)
Working sector	Government	265 (32.7)
	Private	545 (67.3)

The knowledge level of health care professionals towards COVID-19 is presented in Table-II. A majority of participants were familiar with COVID-19 (51.8%) and its etiology (94.5%). While 79.2% of participants were aware of the mode of transmission. 77% participants were aware of the 14 days incubation period. Surprisingly, 81.7% believed that COVID-19 is like a normal flu in sign and symptoms, which is alarming in current circumstances. Among participants, 48.2% believed that COVID-19 virus could live outside the human body from 09 to 48 hours. Regarding which age groups of patients are mostly affected by COVID-19, a majority (78.7%) believed that it affects elderly, however only 7.6% suggested that it affects infants. Among all participants, 69.5% were aware that COVID-19 has 2% mortality rate. Regarding preventive measures of COVID-19, nearly 66% were aware of the preventive methods including, frequent hand washing with soap and use of alcohol-based sanitizer, covering mouth when coughing and sneezing, and wearing masks in public. Lastly, 69.6% considered that the best management option is to isolate at home and practice social distancing measures.

**Table-II:** Knowledge and awareness assessment of health care professionals (n=810).

		Percentage
What is the virus for covid-19?	It is a new strain of virus	51.8
	Mutation in coronavirus	28.9
	Same as MERS COV	4.1
	Its same as SARS COV	6.1
	Don’t know	9.1
What is etiology of covid-19?	Viral	94.4
	Bacterial	3.0
	Genetic	1.5
	Autoimmune	0.5
	Don’t know	0.6
What do you think is the mode of transmission?	Respiratory droplets	14.7
	Shaking hands with infected people	3.6
	Use of objects used by infected people	1.0
	All the above	79.2
	Don’t know	1.0
From where you heard about COVID- 19?	Social media	36.5
	Print media	1.0
	Television	9.1
	Friends and family	3.0
	All the above	46.2
What is the incubation period of COVID-19?	2-14 days	77.2
	3 weeks	10.7
	1 month	5.6
	Don’t know	6.6
Are you aware of the signs and symptoms of COVID-19?	Same as seasonal flu	81.7
	Same as GIT upset	2.5
	Neurological problems	2.5
	All of the above	10.7
	Don’t know	0.5
Are you aware how many days COVID-19 survive outside the body?	9 hours	30.5
	More than 48 hours	10.7
	48 hours	17.8
	less than 24 hours	15.7
	Don’t know	25.4
Which age group is affected the most from COVID-19?	Infants	7.6
	Adolescents	3.6
	Middle age	2.5
	Elderly	78.7
	Don’t know	2.5
Are you aware of the mortality rate COVID-19?	< 2%	69.5
	10%	11.2
	50%	3.0
	90%	2.0
	Don’t know	13.7
What are the ways of prevention from COVID-19?	Washing hands with soap and alcohol-based sanitizer	6.6
	Covering mouth when coughing and sneezing	2.5
	Wear surgical mask in public	4.1
	All the above	65.5
	Don’t know	0.5
What are the treatment options available currently for COVID-19 patients?	Quarantine	34.0
	Vaccines	1.5
	Drugs only	1.0
	Use of preventive measures	39.6
	All the above	16.2
	Don’t know	1.5

Findings regarding attitude and practice of HCPs about COVID-19 are presented in Table-III. About 96.4% were aware that it is contagious in nature and 67% considered themselves updated with management guidelines and however more than quarter of the participants (26.4%) were not aware of the CDC guidelines. Accordingly, 51.3% believed that disease can be transmitted from animals to humans and discouraged them in hospital settings. A large number of participants (43.7%) believe that humans are infected only once and a majority of patients recover from the disease. Within participants, 76.6% of HCPs were aware, that a vaccine is not available hence prevention and basic standards should be followed, however almost a quarter participants either were unaware or thought that there is a vaccine available for SARA-COV-2. 90.9% believed that COVID-19 is a serious threat to the local community if not controlled. Only 7.1% of the participants came across a COVID-19 patient in their workplace. Seventy three percent of participants did not attend any lecture, workshop or seminar on COVID-19 for awareness purpose. 87.8% were practicing washing hands soap and use of alcohol-based sanitizer. Nearly Eighty four percent were using surgical mask when managing general patients, while only 11.6% favored the use of N-95 masks. Similarly, 67.8% of health care professionals were practicing universal precaution for infection control and 57.4% were using sodium hypochlorite as a surface disinfectant in dental surgeries.

**Table-III:** Attitude and practice assessment of health care professionals (n=810).

	Yes %	No %	Do not know %
Covid-19 is contagious and one should contain that?	96.4	2.5	1
Do you think we should prevent the spread as it leads to morbidity and mortality?	28.9	65.0	6.1
Do you think that the virus transmits from animals to humans and vice versa?	51.3	27.3	20.8
Do you know that the virus can affect humans more than once?	43.7	14.7	41.6
Is there any vaccine available?	6.6	76.6	16.2
Do you consider it a threat for our community?	90.9	4.1	4.1
Did you follow CDC guidelines for patients care?	67.0	26.4	5.6
Have you ever come across a patient with COVID-19 patients?	7.1	80.2	11.7
Have you attended any lecture/course/workshop on covid-19?	24.4	73.6	1.0
Do you wash hands before and after contact with your patients?	87.8	2.0	8.1
Do you wear a surgical mask during patient contact?	83.7	5.1	9.6
You wear N-95 mask during patient contact?	11.6	64.0	22.4
Do you follow universal precautions of infection control?	67.8	3.6	7.1
Do you use sodium hypochlorite as surface disinfectant?	57.4	23.4	17.8

## DISCUSSION

COVID-19 infection is spreading worldwide and has become the single most important global concern. Health care professionals who are in close contact with the infected patients play a major role in infection control.^14^ Multiple reports have surfaced regarding the infection and deaths of health care professionals from COIVD-19 infections. Explanations for increasing COVID-19 among HCPs may include, knowledge and practice towards SARS-COV 2, availability of personal protective equipment (PPE), strength and support of the health care system and continued research and its interpretation. Therefore, the present study aimed to investigate the trends in knowledge, awareness and practices amongst health care professionals towards COVID-19 disease control during the outbreak in 2020. The current knowledge and awareness of HCPs as perceived in the study was adequate. However some of the practice requirements observed as a study outcome were compromised. Therefore the hypothesis was partly rejected.

The overall response rate was 81% that is comparatively high in comparison to questionnaire-based studies previously.^15^,^16^ This could be attributed to the immediate concern of the participants (health care professionals) to contribute towards finding solutions to curb the contagious viral pandemic. HCPs routinely take standard precautions considering each patient as infected i.e able to transmit infection. However, considering the highly contagious nature of the SARS-COV 2, and in some cases asymptomatic occurrence, the usual daily protective measures taken may not be effective and extreme strict cross infection protocols are recommended. In the present study among HCPs, 96.4% were aware that COVID-19 is contagious in nature. This finding is in line with a research on Iranian nurses, where 94.11% believed it to be contagious.^14^ This study found that 67% of the respondents were updated and practiced CDC guidelines for all patients. This improvement in precautionary measures are found in similar studies.^17^ However, poor knowledge levels persist amongst health care workers as shown in a previous study and greater efforts are required to create and spread awareness.^18^ Furthermore 90.9% considered the virus to be a problem for our community. This perception was reflected by 67% of the participants who practiced CDC guidelines for all patients. But nearly quarter participants did not practice the CDC guidelines, putting themselves and others at risk of infection. A greater emphasis is also required on use of surface disinfectants by the health care workers, as only 57% of HCPs practiced the use for hydrogen peroxide for disinfection of the virus. Hydrogen peroxide vapor is an effective viricidal agent for structurally distinct viruses dried on surfaces^19^ and is recommended along with 70% ethanol and quaternary ammonium compound for viral disinfection.^20^

The contagious nature of the virus warrants the existence of sound knowledge regarding incubation period and symptoms amongst the health care workers. In the present study, 77.2% of the health care professionals believed that COVID-19 has a 2-14 days incubation period. This was in accordance with a similar web-based study amongst the health care workers where 84.3% were found to believe that the symptoms appear in about 2-14 days.^21^ In the same study it was observed, that 44.1% of HCPs had an opportunity to attend a lecture/workshop/ course on COVID-19,^21^ while in our study only 24.4% attended a professional scientific event for COVID-19 update. By contrast a similar study reported 64.63% of the health care professionals completed a COVID-19 training program.^22^ This shows the need of training and courses in our region that can be conducted online to enhance the knowledge of our health care workers. In addition, multiple online webinars are provided by the CDC and world health organization (WHO) for HCPs to access update knowledge in relation to COVID-19. Moreover, 43.7%. participants also felt that there is a possibility of reinfection in previously infected individuals from the virus. However, to our knowledge from indexed literature there is no evidence of re-infection of individuals, and further studies in this regard are warranted. It is also observed the majority of HCPs learned about the facts of COVID-19 from multiple media sources, which is in line with the findings of similar previous studies.^22^ Although it is encouraging to know that information is readily available about the outbreak, however, one has to be very careful about the reliability and authenticity of the source, as misinformation can do more harm than good.

The study presents preliminary findings related to the knowledge and practice of HCPs in association with COVID-19 pandemic in Pakistan. However one should interpret this data cautiously, due to the underlying cultural and contextual factors. In addition the wide variety of participants, may have influenced the outcomes of the study. Based on these critical findings it is suggested that although the majority of HCPs displayed adequate knowledge and awareness, there are aspects of the COVID-19 disease, which still need to be better understood, and improvements in the practice of HCPs when managing patients in the COVID-19 pandemic are recommended. The rapidly spreading COVID-19 has become a challenge for health care professionals who are in close contact with patients in decreasing the spread by means of efficient use of preventive measures.^23^ All hospitals and clinics should establish pre-check triages for all the patients as well as the staff; patients should be asked regarding their health status and travel history. Besides that, temperature of the patients and their attendants should be evaluated once they are in the hospital premises; if the patient has travel history within past two weeks, they must be placed in quarantine for a minimum of 14 days. Patients with continuous high fever along with any other symptom including, sore throat persistent cough, head or body aches, diarrhea should be registered as a COVID-19 patient until proven otherwise and referred to the designated hospital facilities.^24^ All elective hospital medical, dental and surgical procedures should be deferred except emergencies, and use of recommended PPEs including N-95 masks must be practiced.^25^ Disinfection of the premises and instruments with hydrogen peroxide and/or 70% Ethanol before each emergency procedure is recommended.^25^ Continued education program including seminars, webinars and courses, on updated findings related to COVID-19 infection prevention, modes of transmission and management protocols for the HCPs must be made mandatory. In addition, there is a need for continued medical, financial and psychological support for the health care professionals for the continued risks they are exposed daily in battling the COVID-19 pandemic. Further studies are recommended to identify the reasons for reported high infection rates of HCPs.

## CONCLUSION

The study suggests that the vast majority of the health care professionals have adequate knowledge and awareness related to COVID-19. However, some aspects of practice of health care professionals were found to be deficient including, following CDC guidelines during patient care, acquiring verified knowledge related to COVID-19, disinfection protocol and the use of N-95 mask. Mandatory Continued professional development programs including lectures and workshops on COVID-19 for all health care professionals are the need of the hour, to manage the pandemic and limiting the morbidity and mortality related to it.

### Authors’ Contribution

**NA:** Data collection, study design, manuscript writing, final manuscript approval.

**MS:** Data collection, study design, manuscript drafting, data analysis, manuscript approval.

**FV:** Questionnaire design and preparation, Data collection, manuscript approval, data interpretation and is responsible for integrity of research.

**TA:** Data collection, writing, revise, editing and final manuscript approval.

**QM:** Study design, statistical analysis, data interpretation, manuscript writing, table and figure designing.

**MAR:** Statistical analysis, Manuscript writing, Editing of manuscript.
